# Effectiveness of Early Physiotherapy in an Infant With a High Risk of Developmental Delay

**DOI:** 10.7759/cureus.16581

**Published:** 2021-07-23

**Authors:** Namrata Sant, Rinkle Hotwani, Pallavi Palaskar, Waqar M Naqvi, Sakshi P Arora

**Affiliations:** 1 Physiotherapy, Mahatma Gandhi Mission (MGM) School of Physiotherapy, Aurangabad, IND; 2 Physiotherapy, Mahatma Gandhi Mission (MGM) Institute of Health Sciences, Navi Mumbai, IND; 3 Physiotherapy, Ravi Nair Physiotherapy College (RNPC), Wardha, IND

**Keywords:** prematurity, early intervention, oromotor stimulation, sensory integration, delayed developmental milestones, premature birth, neurodevelopmental treatment

## Abstract

Premature birth is the most common cause for a stay in the neonatal intensive care unit (NICU) among neonates. Premature birth leads to prematurity, which is associated with complications such as respiratory distress syndrome (RDS), hyperbilirubinemia, gastroesophageal reflux (GERD), intraventricular hemorrhage (periventricular leukomalacia), retinopathy of prematurity (ROP), and so on. These secondary complications are of great concern and need to be handled with care to prevent the further deterioration of the quality of life of the baby as he grows. So, the early physiotherapeutic interventional approach comes into light and plays an important role in neonatal care.This case study demonstrates an infant boy of seven months chronological age, who had a preterm birth history with a poor APGAR (Appearance, Pulse, Grimace, Activity, and Respiration) score and NICU stay during the first three months of life. He approached the physiotherapy outpatient department with a complaint of delayed motor milestones like an absence of head holding, rolling, opening of hand, as well as delayed social-emotional development, Ryles tube (RT) in situ, with frequent episodes of GERD, neck rotated to the left side, high irritability, tactile defensiveness of both hands, and difficulty in regulating his sensory systems. Outcome measures used were gross motor function measure (GMFM), sensory profile, and rotating chair test. Early interventional physiotherapy was given including neurodevelopmental techniques (NDT), oromotor stimulation, sensory integration, passive stretching, and myofascial release for six days per week with each session of 45 minutes. The results demonstrated the achievement of motor milestones till sitting independently, reduced episodes of GERD, discontinued RT in situ, improved mobility of neck on both sides, reduced irritability, and started reaching, grasping along with bimanual tasks.

## Introduction

Medical and technological advances in neonatal care during the mid to late 1900s led to the decreased mortality rate in neonates and increased the survival rate of very low birth weight infants resulting in increased incidences of cerebral palsy, respiratory disorders, blindness, cognitive delays, and hearing impairments globally [1]. Early intervention programs have been established to address the developmental needs of these survivors of neonatal intensive care, with the goals of stimulating and normalizing their development [2]. The philosophy of management of infants in NICU had changed over a period of time from minimal handling to early stimulation of these infants [3].

Premature birth leads to prematurity, which is the commonest cause of neonatal intensive care unit (NICU) stay of infants. The prematurity is associated with medical complications like respiratory distress syndrome (RDS) [4], hyperbilirubinemia [5], gastroesophageal reflux (GERD) [6], intraventricular hemorrhage (periventricular leukomalacia) [7], retinopathy of prematurity (ROP), and so on. These secondary complications are of great concern and need to be handled with care to prevent the further deterioration of the quality of life of the baby as he grows [8]. So, the early physiotherapeutic interventional approach comes into existence and plays an important role in neonatal care.

The term ‘early intervention’ refers to the multidisciplinary services provided to children from birth to six years of age to promote child’s health and well-being, enhance emerging competencies, minimize developmental delays, remediate existing disabilities, prevent functional deterioration, and promote adaptive parenting and family functioning [9]. So, this case study evaluated the effect of early intervention on high-risk infants with delayed developmental milestones along with longer NICU stay.

## Case presentation

This report is reporting a preterm infant boy (28th week of gestation) with the chronological age of seven months and a corrected age of 5.5 months. The mother of the infant had conceived by the in-vitro fertilization (IVF) twin pregnancy procedure. The mother presented to the inpatient department with cervical opening and intrauterine contraction at the time of the 28th week of pregnancy and hence underwent emergency cesarean section and gave birth to twin baby boys. The twin infant was developmentally appropriate for his chronological age. The weight of the infant at birth was 870 grams and his head circumference was 27 cm, he did not cry immediately after birth, APGAR (Appearance, Pulse, Grimace, Activity, and Respiration) score was 5 at 1 min and 7 at 5 min. He was admitted to the NICU during the first three months of life during which he was on ventilatory support for the first 15 days and later shifted on continuous positive airway pressure (CPAP) and oxygen support for the remaining days of NICU stay. During his admission, he underwent surgery for an inguinal hernia at 2.5 months of age, following which he went through aspiration pneumonia, which was diagnosed as a case of gastroesophageal reflux disease (GERD) and managed by Ryles tube (RT) in-situ.

**Figure 1:**
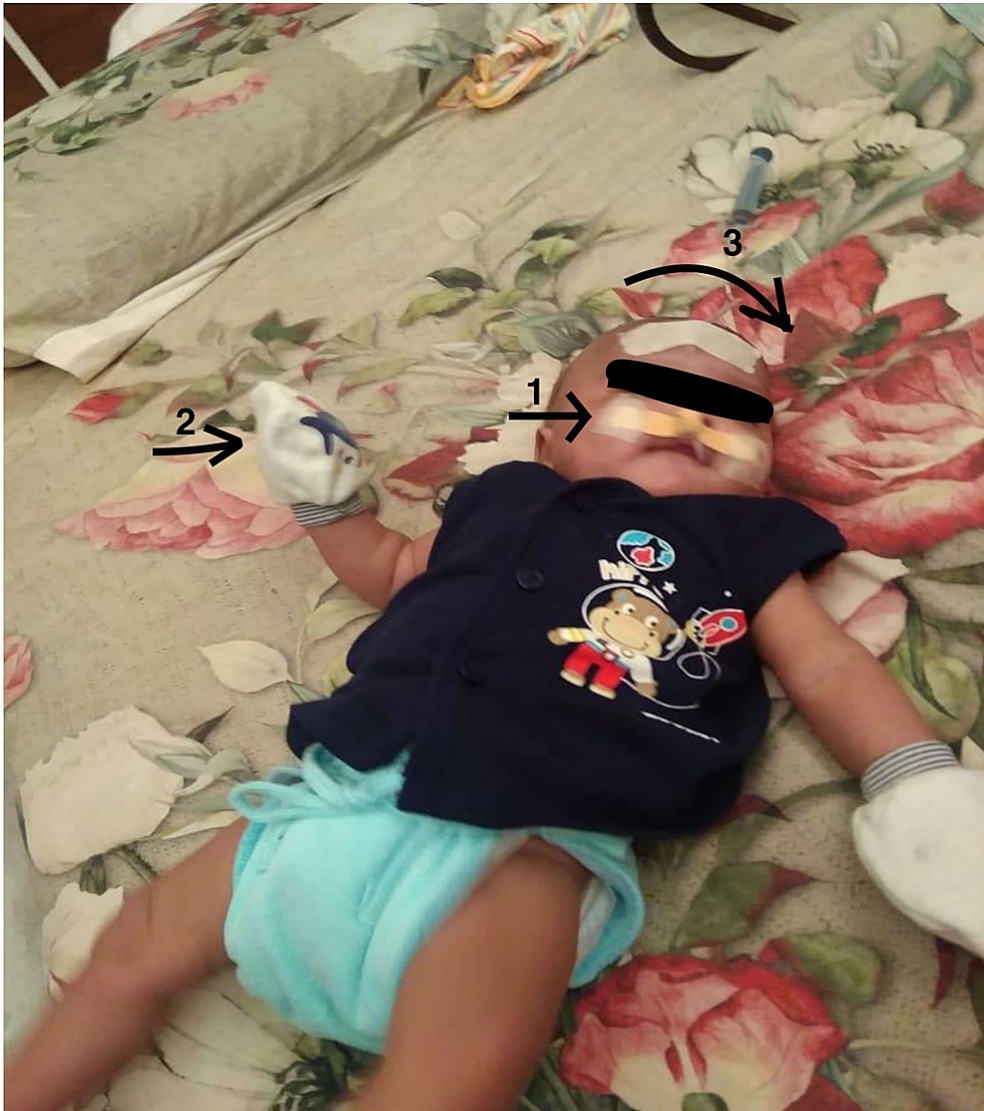
Demonstrating 1: RT in situ; 2: Tactile defensiveness for both hands: and 3: Positional torticollis RT: Ryles tube

At the physiotherapy OPD, the infant was presented with delayed motor milestones like the absence of head holding, rolling, opening of hand as well as delayed social-emotional development, RT in situ, with frequent episodes of GERD, neck rotated to the left side, positional torticollis, high irritability, difficulty in regulating sensory systems, and tactile defensiveness of both hands as shown in Figure 1.

The physiotherapeutic approach for the baby followed neurodevelopmental techniques, oromotor stimulation, sensory integration, and passive stretching with myofascial release. The details of each intervention following the FITT (frequency, intensity, time, and type) principle are described in Table 1. The effectiveness of the treatment techniques followed was analyzed using the gross motor function measure (GMFM) score, removal of RT in-situ, sensory profile, and rotating chair test in torticollis. The details of the pretreatment and post-treatment scores of each outcome measure are demonstrated in Table 2.

**Table 1:** Physiotherapeutic intervention plan followed for the infant

Intervention	Frequency (F), Intensity (I), Time (T), and Type (T)
Neurodevelopmental techniques [10]	F: 6 days/week T: 45 minutes of the session Facilitation of head holding on a wedge or swiss ball, rolling and obliques on the mat, neck extension on swiss ball prone with scapular retraction, rolling with a caudal weight shift to the rib cage, intercostal muscle stretch progressing towards pull to sit on the mat, side-sitting one hand weight-bearing with asymmetric weight shift, prone on elbows and hands-on swiss ball, sitting on tilt board with weight shifts, and sitting 90-90 upright on the small stool.
Oromotor stimulation [11]	The devices used were finger brush with soft bristles, progressing to vibrating brush and nuke brush, the position of the head in midline, slightly flexed with the propped up position. The techniques used included were stimulation of intra-oral and peri-oral structures with a gloved finger, stroking over cheeks to improve sucking rate, deep pressure on gums with a gloved finger, intraoral stimulation with artificial nipples, and cue-based peri-oral sucking and rooting reflex facilitation The dosimetry included 20 mins before breastfeeding on RT tube, 5-10 mins depending on signs of stability and stress with a frequency of before each and every feed. Sequential introduction of different tastes like breast milk, honey, jaggery, sugar (fine/coarse).
Sensory integration (SI) [12]	The tactile system stimulation followed stroking of palms with different textures from soft to hard, exposure to all types of clothing, blankets, and toys. The vestibular system stimulation included head nod with 20 repetitions, head turns with 20 repetitions, swing on the horizontal plane in the supine position in forward, backward and lateral direction, cribbing in the vertical plane in supine, and rolling on the swiss ball. The progression included bouncing on the swiss ball in a sitting position and sitting on a rotating swing. The proprioceptive system stimulation included techniques like tightly and firmly rolling into the blanket, joint compression concentrating more on all joints of extremities, vibrations using tunning-fork or brush, giving hugs, and using weighted blankets. Further progression followed giving firm deep pressure using the large ball to ‘steam roll’ over the child’s body.
Passive stretching with the myofascial release [13]	The passive stretching followed two techniques in which technique 1 included stabilization of upper part of thorax with one hand, laterally flexing the head of the child to the left side with 10 seconds hold and then laterally rotating the child’s head to the right side with a 10 sec hold to stretch sternocleidomastoid. The second technique used was in terms of the home stretching program of technique 1 with the use of a rotating chair as per the convenience of the caregiver. The stretches followed three repetitions per exercise for six weeks. Also, the tummy time, i.e. the time spent in the prone position was increased for 30 to 60 minutes/day following the myofascial release.

**Table 2:** The outcome measures demonstrating the pretreatment and post-treatment status of the infant where NT is not tested according to the criteria of the scale used NT: not tested

Outcome Measure	Pre-score	Post-score
GMFM score [14]	Lying & rolling was 9.81; Sitting was 0; Crawling and kneeling were NT; Standing was NT; Walking, running, jumping was NT giving the total score of 1.97%. (where NT is not tested)	Lying & rolling is 84.3; Sitting is 18.34; Crawling and kneeling were NT; Standing was NT; Walking, running, jumping was NT making the total score 20.53%.
Presence of RT in-situ	RT in situ, gag reflex was absent, 7-8 episodes of Reflux /day, and oral feed was absent.	RT got removed, the gag reflex is present, no episode of reflux/day, able to consume semi-solid food, liquid food is non-compatible more than 1 spoon at a time.
Sensory profile [15]	In quadrants, seeking was 35 (seeker), avoiding was 43 (defensive), sensitivity was 38 (detects sensory input at a high intensity), and registration was 21 (misses out sensory input at a high intensity). The sensory and behavioral section included a general score of 23, an auditory score of 20, a visual score of 24, a touching score of 31, a movement score of 20, an oral score of 21, and a behavioral score of 22. All of the components fall into the “more than others” classification system of sensory profile.	In quadrants, seeking is 31 (normal), avoiding is 12 (normal), sensitivity is 17 (normal), and registration is 14 (normal). The sensory and the behavioral section includes a general score of 13, auditory score as 8, visual score as 20, touch score as 12, movement score as 15, oral score as 11, and behavioral score as 8. All of the components fall into the “just like the majority of others” classification system of the sensory profile, except visual, there is no change.
Rotating chair test- Torticollis [16]	Decreased mobility towards right neck rotation.	Complete mobility of cervical rotation.

**Figure 2:**
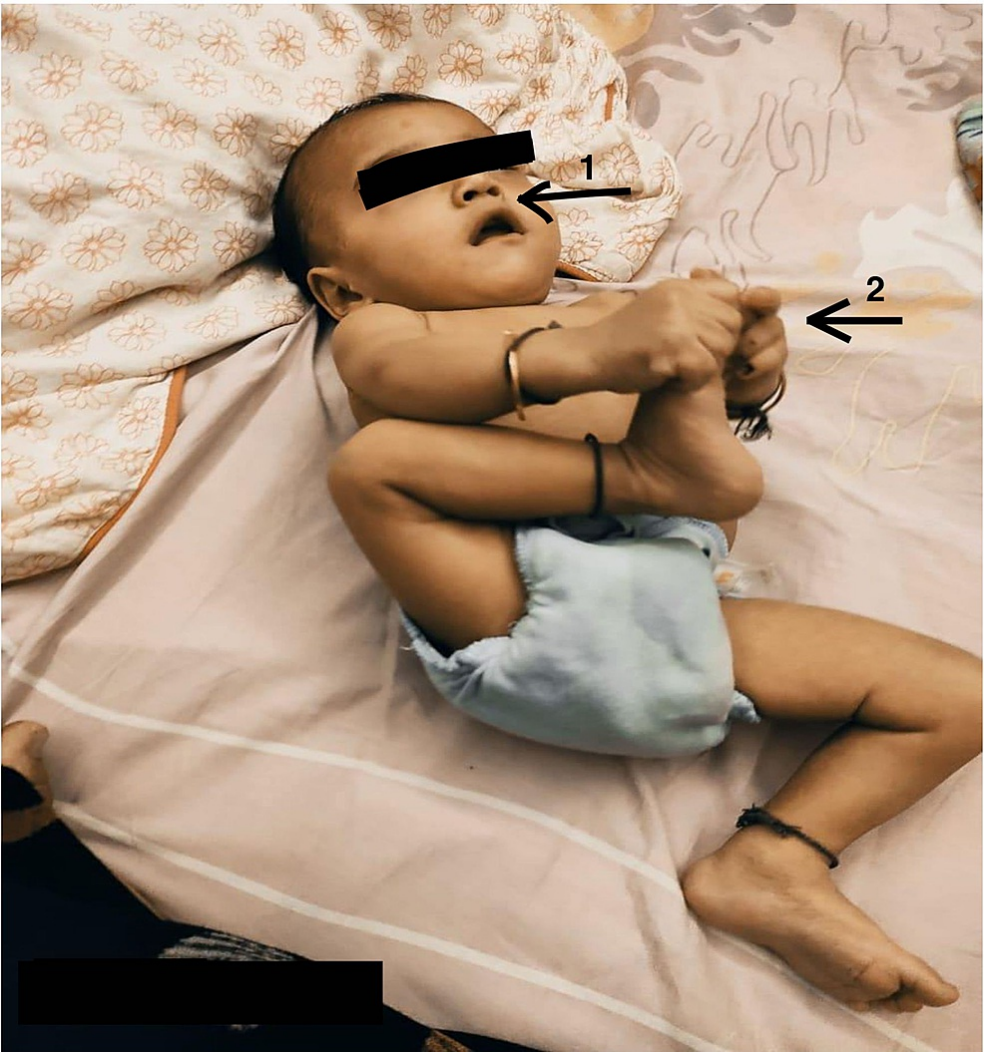
Demonstrating 1: RT in situ removed; 2: Presence of grasping with no tactile defensive aid RT: Ryles tube

## Discussion

Preterm birth is a risk factor for developmental delay. This study identified the developmental risk and carried out the necessary physiotherapy treatment within the framework of early intervention studies. There’s an ample amount of literature available on the effectiveness of individual techniques of physiotherapy but this report has concentrated on the precision of the physiotherapy treatment protocol according to the patient’s need. Hence, the effective combination of neurodevelopmental techniques, oromotor stimulation, sensory integration, and passive stretching with the myofascial release was followed for the presenting patient. When pre and post-outcome measures were compared, it has been found that the infant has achieved a majority of motor milestones: started having semi-solid oral food in place of RT in-situ, started grasping for different textured toys, midline play with bimanual activity, improved visual tracking on the right side, decreased irritability, improved regulation, and started exploring the environment around him as shown in Figure 2. These results suggest that the applied early intervention study had a positive effect on the developmental outcome of infants.

Neuroplasticity, i.e., the neural tissue's ability to generate and reorganize the synaptic connections specifically to learning or following any injury, may be the possible reason for the positive outcome of early intervention therapy. An infant’s brain is not in a static phase, but it has the potential to change with different environmental cues, which may be the basis for neuro-rehabilitation. Brain development is proved to be dynamic at an early stage of life. According to literature, neonates in NICU are capable of responding to the internal and external changing environments [17].

Early intervention is inclusive of the facilitation of neuromotor, sensory, oromotor, musculoskeletal, and social, language milestones. Neuro-developmental techniques facilitate normal posture and movement and hence help in the achievement of further growth and development of neonates [18].

When all the results are evaluated, it can be considered that the combination of early intervention programs applied within the scope of the study served its purpose and has had a positive effect on all the individuals who are served with therapy.

## Conclusions

This case report has reported the effectiveness of the combination of the physiotherapeutic treatment techniques of neurodevelopmental techniques, oromotor stimulation, sensory integration, and passive stretching with myofascial release. The infant’s achievement of the majority of developmental milestones, being able to consume semi-solid food and getting rid of RT in-situ, identification and activity independence for grasping different textured toys, improvement in bimanual activities, improved visual response to the point of stimulation, improved behavior, and improved regulation and exploration of environment around him, made a significant demonstration on the effectiveness of the physiotherapeutic approach in preventing the delayed milestone complication in high-risk infants. The application of early intervention has been explored with an effort towards creating a new line of treatment for the increasing incidence of preterm birth complications.
